# Fitness Analysis and Transcriptome Profiling Following Repeated Mild Heat Stress of Varying Frequency in *Drosophila* *melanogaster* Females

**DOI:** 10.3390/biology10121323

**Published:** 2021-12-14

**Authors:** Nataly E. Gruntenko, Evgenia K. Karpova, Vladimir N. Babenko, Gennady V. Vasiliev, Olga V. Andreenkova, Margarita A. Bobrovskikh, Petr N. Menshanov, Roman O. Babenko, Inga Yu. Rauschenbach

**Affiliations:** 1 Institute of Cytology and Genetics SB RAS, 630090 Novosibirsk, Russia; karpova@bionet.nsc.ru (E.K.K.); bob@bionet.nsc.ru (V.N.B.); genn@bionet.nsc.ru (G.V.V.); andreenk@bionet.nsc.ru (O.V.A.); eremina@bionet.nsc.ru (M.A.B.); eternity@bionet.nsc.ru (P.N.M.); rbab@bionet.nsc.ru (R.O.B.); iraushen@bionet.nsc.ru (I.Y.R.); 2 Physiology Department, Novosibirsk State University, 630090 Novosibirsk, Russia; 3 Laser Systems Department, Novosibirsk State Technical University, 630073 Novosibirsk, Russia

**Keywords:** longevity, fertility, heat stress, metabolism, transcriptome, *Turandot*, *Hsp23*

## Abstract

**Simple Summary:**

We studied the effect of mild heat stress (38 °C, 1 h) occurring once a day or once a week on *D. melanogaster* fertility, longevity, body composition metabolism and differential gene expression in fat body and adjacent tissues. Weekly stress in the first two weeks did not affect longevity but caused a decrease in fat content and an increase in the total level of fertility. Daily stress caused a significant longevity, fertility and fat content decrease, but an increase in carbohydrate levels compared with the control group. These data agree well with the results of transcriptome analysis, which demonstrated significant changes in expression levels of genes involved in proteolysis/digestion following daily stress. Heat shock protein 23 and stress-inducible humoral factor *Turandot* gene network are also involved. It is notable that daily and weekly heat stress resulted in different changes in metabolism, fitness and differential gene expression.

**Abstract:**

Understanding how repeated stress affects metabolic and physiological functions in the long run is of crucial importance for evaluating anthropogenic pressure on the environment. We investigated fertility, longevity and metabolism in *D. melanogaster* females exposed to short-term heat stress (38 °C, 1 h) repeated daily or weekly. Daily stress was shown to cause a significant decrease in both fertility and longevity, as well as in body mass and triglyceride (fat) content, but a significant increase in trehalose and glucose content. Weekly stress did not affect longevity and carbohydrate metabolism but resulted in a significant decrease in body mass and fat content. Weekly stress did not affect the total level of fertility, despite sharp fertility drops on the exact days of stressing. However, stressing insects weekly, only in the first two weeks after eclosion, caused a significant increase in the total level of fertility. The analysis of differentially expressed genes in the fat bodies and adjacent tissues of researched groups with the use of RNA-Seq profiling revealed changes in signal pathways related to proteolysis/digestion, heat shock protein 23, and in the tightly linked stress-inducible humoral factor *Turandot* gene network.

## 1. Introduction

All living organisms are exposed to unpredictable temperature fluctuations and other climate-dependent unfavourable conditions throughout their life. Under fluctuating environmental conditions, a number of the organism’s adaptive responses are launched on cellular, humoral and behavioural levels. These adaptive responses ensure the effective regulation of reproduction and ageing under harsh environmental conditions in most animals, including an important model organism *Drosophila melanogaster* (Diptera: Drosophilidae). In *D. melanogaster*, stress response involves the heat shock protein network [1], the c-Jun-N-terminal kinase (JNK) signalling pathway [2,3], the stress-inducible humoral factor *Turandot* gene network [4], the insulin/insulin-like growth factors signalling pathway and complex changes in neurohormonal status, including biogenic amines, 20-hydroxyecdysone, and adipokinetic and juvenile hormones [5,6,7,8,9]. Neurohormonal response to stress is known to produce a decrease in fertility level in insects as a result of oviposition arrest caused by juvenile hormone and the resorption of early vitellogenic oocytes induced by 20-hydroxyecdysone [10]. Thus, an acute response to unfavourable conditions is rather well described, whereas much less is known concerning the effects of multiple stressors and their delayed consequences. Moreover, suboptimal increases in environmental temperatures become more common due to anthropogenic global warming [11]. However, little is known about possible cumulative effects of heat stresses experienced by animals during their life.

There is evidence that stress can have both negative and positive effects on *D. melanogaster* fitness [12]. In particular, it was shown that heat stress could increase or decrease longevity depending on the age and genetic background of flies [13,14] and heat exposure duration [15]. Heat shock procedures were also found to be able to increase *Drosophila* resistance to acute heat stress [15,16,17,18]. We previously demonstrated that increased survival rate under acute heat stress following mild heat stress (38 °C, 1 h) repeated daily within 2 weeks correlates with increased activity of the dopamine metabolism enzymes, dopamine-dependent arylalkylamine N-acetyltransferase and alkaline phosphatase [18], which suggested a decrease in dopamine level [19]. We believe that this decrease could possibly contribute to adaptation, allowing insects to save energy, as dopamine is known to regulate energy metabolism, foraging and locomotion in insects [20,21,22,23]. The most important energy store of the insect organism is the fat body, a key organ in the metabolism of lipids, carbohydrates and vitellogenins, as well as juvenile hormone, which acts as a growth regulator in larva and as a gonadotropin in adults [24,25,26,27]. In the fat body, carbohydrates are stored in the polymeric form of glycogen, which can be quickly degraded to glucose and trehalose on demand to be used as a glycolytic fuel; lipids are stored as triglycerides, form the long-term energy reserve, and can also be used by insects for energy production through β-oxidation to meet their demands [25].

In the present study, we examine the long-term consequences of repeated stress exposure (model of frequent natural situations) in *D. melanogaster*. We found that exposure to mild heat stress (38 °C, 1 h) causes different effects on fitness, metabolism and differential expression of genes in fat bodies and adjacent tissues, depending on whether it was repeated daily or weekly. We consider 1 h exposition in a 38 °C air incubator to be a mild stress in the context of this study, because earlier we demonstrated that flies did not stop the oviposition and their ecdysteroid content and juvenile hormone degradation did not change if the stress exposure is 2 h or less [10,28]. Male fertility was also not disturbed by 1 h heat stress at 38 °C [29].

## 2. Materials and Methods

### 2.1. Drosophila melanogaster Strain and Heat Stress Modes

Flies of the wild type *D. melanogaster* strain Canton S were maintained on a standard medium (agar-agar, 7 gL^−1^; corn grits, 50 gL^−1^; dry yeast, 18 gL^−1^; sugar, 40 gL^−1^) at 25 °C. Flies were synchronized at eclosion (flies that eclosed within 3 to 4 h were collected), and experimental groups were exposed to heat stress on a daily or weekly basis.

The stress exposure was performed by transferring vials with flies (five females and five males per vial at the start of experiment) from a 25 °C incubator to a 38 °C incubator for 1 h in the 24 h after eclosion and once a day after that for the “daily stress” group; in 2 days after eclosion and every 7 days after that for the “weekly stress” group; in 2 and 9 days after eclosion for the “starting weekly stress” group. In fertility experiments, flies were transferred to the fresh medium daily, and three times a week in all other experiments. In the longevity experiments, flies of the “daily stress” group were exposed to 38 °C for 1 h five days a week. In the fertility and longevity experiments, flies were maintained until the end of reproductive period and life, respectively. In all the other experiments, flies were analysed on the 12th day after eclosion (24 h after the heat stress exposure for the “daily stress” group and 72 h after it for the “starting weekly stress” group). For fat and carbohydrates content measurement, 12-day-old females were frozen in liquid nitrogen and stored at −20 °C. For RNA isolation, 12-day-old females were dissected in Ringer’s solution and their abdomens without internal organs were also frozen in liquid nitrogen. This part of fly body is addressed here as “fat body and adjacent tissues”.

### 2.2. Fertility and Longevity Analysis

For fertility analysis, five newly eclosed females and five males were placed into a vial (10 vials per group) and were transferred to vials with fresh medium daily. Fertility was expressed as the number of adult offspring per female per 24 h with the use of the SeedCounter mobile application [30]. All the flies hatched within 5 days in each vial were counted. For longevity analysis, five newly eclosed females and five males were placed into a vial (10 vials per group) and were transferred to vials with fresh medium three times a week. The daily survival rate (DSR) was calculated every day as a proportion of flies alive per day to a total number of flies enlisted in the experimental group initially.

### 2.3. Body Mass and Triglyceride Content Measurements

For body mass evaluation, individual females from one control and two experimental groups (9–11 flies for each group) were weighed at the 12th day after eclosion. Triglyceride content was evaluated with the use of spectrophotometric assay described by Mukherjee and Mishra [31]. Flies were merged into groups of 10 individuals to obtain a sufficient amount of triglycerides in a sample (4–6 samples were measured for each group under study). Decapitated flies were homogenized on ice in PBST buffer (0.2% Tween-20 в 1X PBS) and centrifuged for 5 min at 3075 g. Supernatant was transferred into a microcentrifuge tube, which was then heated at 70 °C for 10 min. Then 20 μL of PBST or 20 μL of Triglyceride reagent (Sigma-T2449) was added to 20 μL of supernatant or PBST blank, or glycerol standard in individual tubes. The tubes were incubated at 37 °C for 1 h and then centrifuged for 3 min at 17,709 g. 30 μL samples from each tube were taken to a 96-well plate, 100 μL free glycerol reagent (Sigma-F6428) was added to individual well, and the plate was incubated at 37 °C for 5 min. The absorbance was measured with the use of a Multiscan SkyHigh spectrophotometer (Thermo Fisher Scientific, USA) at the wavelength of 540 nm. To measure the triacylglyceride concentration, the absorbance of free glycerol in the untreated sample without Triglyceride reagent was subtracted from the concentration of total glycerol of samples treated with Triglyceride reagent.

### 2.4. Carbohydrates Metabolism Evaluation

Carbohydrate metabolism in females was evaluated with the use of a SmartSpec Plus spectrophotometer (Bio-Rad, Hercules, CA, USA) at the wavelength of 340 nm as described earlier [32]. Decapitated flies were homogenized in hypotonic buffer for lysis (20 мM HEPES, 2 мM MgCl_2_, 2 мM EGTA) and then were placed into cooled microcentrifuge tubes on ice. After 10 min of incubation, tubes were centrifuged for 5 min at 13,400 g and titers of metabolites were determined in supernatant. Glucose titer was measured using a Glucose (HK) Assay Kit (Sigma-SLBL3912V). Trehalose was converted into glucose by adding trehalase (Sigma, 0.5 units/mL) with a further measurement of glucose titer.

### 2.5. Capillary Feeding Assay

The amount of food consumed by the flies was evaluated using the method of Ja et al. [33] modified by Williams et al. [34]. Five mated 12-day-old females of each group were placed in a vial, 10 cm × 2 cm (height × diameter), containing 1% agarose (5 cm high), which provided moisture and humidity for the flies during the experiment. A capillary glass tube (Narishige, Japan) was filled with 15 μL of liquid food, which contained 5% sucrose and 5% yeast extract, and was put in a vial stopper using two pipet tips (one being placed into another). The initial food level in the capillary tube was marked and 0.1 μL of mineral oil was used to prevent liquid food from evaporating. The vials (4 to 5 for each group) were kept in a 25 °C incubator for 24 h, and then the final food level in the capillary tube was marked to determine total food intake per day. The “blank” vial without flies was used to detect the rate of food evaporation. The average feed consumption of a fly was calculated by dividing the total food intake (minus the “blank” value) by the number of flies in the vial. Each experiment was performed with three biological replicates.

### 2.6. Statistical Analysis of Fitness and Body Composition Data

All data are presented as means ± SEM. The data on fertility (number of offspring per day per female) were analysed via two-way mixed-design ANOVA (with day after eclosion as the within-subjects factor and stress as the between-subjects factor). The data on body weight, feed consumption, fat and carbohydrates contents were analysed via one-way ANOVA (with stress as the between-subjects factor). The comparison of the group means in ANOVA was performed with the Benjamini–Hochberg stepwise post-hoc test. The data on longevity were analysed via Kaplan-Meier method followed by the log-rank test [35]. The false discovery rate corrections for multiple comparisons were made when appropriate. The results were considered significant at a probability level <0.05.

### 2.7. RNA Isolation, cDNA Library Construction and RNA Sequencing

Three (for the control group) to four (for groups exposed to short-term heat stress daily or weekly) independent biological replicates were obtained by RNA extraction from the fat bodies and adjacent tissues of 50 females for each group. For sample preparation, 1 µg of RNA per sample was used as input material. DNA contaminations were removed from the samples with a TURBO DNA-free kit (Ambion, Austin, TX, USA). Total RNA quality was assessed using an Agilent 2100 Bioanalyzer (Agilent Technologies, Santa Clara, CA, USA) with an RNA 6000 Nano Kit (Agilent Technologies, Santa Clara, CA, USA). The mRNA fractions were isolated and the barcoded RNA-Seq libraries for the Illumina system were constructed with a TruSeq Stranded mRNA Library Preparation Kit (Illumina, San Diego, CA, USA) according to the manufacturer’s instructions. The quantity and quality of the libraries were assessed using a DNA High sensitivity kit (Agilent Technologies, Santa Clara, CA, USA) and an Agilent Bioanalyzer 2100 System (Agilent Technologies, Santa Clara, CA, USA). The libraries were then sequenced using an Illumina NextSeq 550 with 75-bp read length and sequencing depth of 20 million reads per library.

### 2.8. Mapping Reads onto the Genome and FPKM Evaluation

The raw reads from ChIP-seq and RNA-seq experiments were trimmed for quality (phred ≥ 20) and length (bp ≥ 32) using Trimmomatic v. 3.2.2 [36]. Illumina adapters were trimmed.

We used STAR software [37] to map the reads onto the DM6.22 genome located at: ftp://ftp.flybase.net/releases/current/dmel_r6.22/fasta/dmel-all-chromosome-r6.22.fasta.gz (accessed on 30 March 2021). Gene annotation was retrieved from: ftp://ftp.flybase.org/genomes/dmel/current/gtf/dmel-all-r6.22.gtf.gz (accessed on 30 March 2021). Overall “clean” coverage was around 18 mln reads per sample; the average number of genes per sample covered was about 27,300 (see below).

### 2.9. Transcriptome Data Analysis

We used Cufflinks software [38] to assess Fragments per million per kilobase (FPKM) expression rate across annotated genes. Eleven sets were processed in three groups: Control (three replicas), day (four replicas), week (four replicas). Cuffnorm software [38] was employed for expression rate assessment in FPKM units and for alternatively spliced transcripts expression profiles reconstruction. A total of 34,600 transcripts were in the reference (annotation) set. Non-zero expressed transcripts in each group were: 27,400 in the “day” group, 27,800 in the “week” group, and 27,300 in the “control” group.

### 2.10. Quantitative Real-Time Polymerase Chain Reaction

For quantitative real-time-polymerase chain reaction (qRT-PCR) analysis, total RNA was extracted from the fat bodies and adjacent tissues of 12-day-old Canton-S females (25 flies per sample for each biological replicate) using a TRIreagent #BCBT8883 (Sigma, USA) according to the manufacturer’s instructions. Synthesis of cDNA was carried out using oligo (dT) 18 priming and a RevertAid First Strand cDNA Synthesis Kit # K1621 (Thermo Scientific, USA). The expression of three *Turandot* genes was analysed on a CFX96 (Bio-Rad, USA) by qRT-PCR using three replicates for every sample. The data were normalized against RpL32. The qPCR mix contained R-402 c SYBR-Green I. (Syntol, Russia) and one of the following primers sets: *RpL32*, CAGCATACAGGCCCAAGATC and CGATGTTGGGCATCAGATACTG; *TotC*, CAGTTTGTCTTAAACCAGTGCTC and CAGATTCCCTTTCCTCGTCAG; *TotA*, AATTCTTCAACTGCTCTTATGTGCT and CAGCAATTCTAAGGTTGTCAGC; *TotM*, AAGCCTGCACTATGAATCCTACAA and CTCATCTTCGTTCTCAGCATTTA; *Hsp23*, TCACTTTGTCCGCCGCTATG and ATGCGCTCGTTGCCCTTATC. Each reaction was performed in triplicates with three biological replicates.

## 3. Results

### 3.1. The Effects of Repeated Mild Stress Episodes of Varying Frequency on Fitness

To evaluate the effect of repeated mild stress episodes on the fitness of *D. melanogaster* adults, we studied the influence of short-term heat stress (38 °C, 1 h) of varying frequency on fertility and longevity of the wild-type strain Canton S.

**Figure 1:**
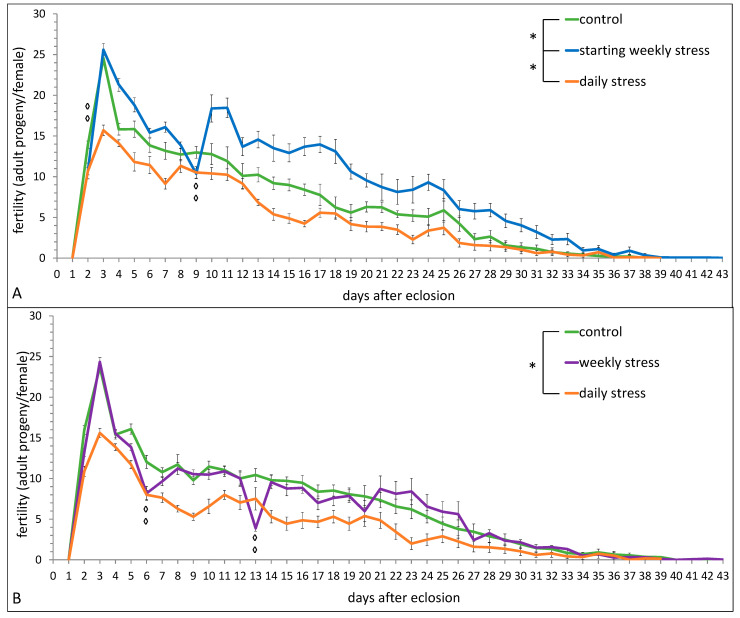
The effect of repeated episodes of mild heat stress (38 °C, 1 h) (**A**) once a day during the whole period of reproduction (daily stress) or once a week within the first 2 weeks (starting weekly stress); (**B**) once a day or once a week during the whole period of reproduction (daily stress and weekly stress, respectively) on the fertility of *D. melanogaster* wild type strain Canton S. Each value is an average of 10 tests. Means ± SEM. The asterisk indicates significant differences between stressed and control flies during the whole period of reproduction [one asterisk, *p* < 0.05, Benjamini–Hochberg stepwise post-hoc test]; the diamond indicates significant differences between weekly stressed and control flies (**A**) on days 2 and 9 (**B**) on days 6 and 13 [two diamonds, *p* < 0.01, Benjamini–Hochberg stepwise post-hoc test].

Figure 1 presents the data on the fertility level of flies exposed to stress daily, on days 2 and 9 after eclosion (“starting weekly stress”), or weekly (on days 6, 13, 20, 27 after eclosion) in comparison with unstressed control.

The fertility of daily stressed flies was significantly lower than that of control flies during the whole period of reproduction (Figure 1A: stress–F_(2,27)_ = 16.36, *p* = 0.00003; age–F_(37,999)_ = 183.17, *p* ≪ 0.0001; stress*age–F_(74,999)_ = 2.78, *p* ≪ 0.0001; Figure 1B: stress–F_(2,27)_ = 5.72, *p* = 0.009; age–F_(37,999)_ = 141.53, *p* ≪ 0.0001; stress*age–F_(74,999)_ = 1.66, *p* = 0.0006). The fertility of weekly stressed flies was comparable with the control group in most days of the experiment, except the exact days of stressing (Figure 1A, stress–F_(1,18)_ = 6.85, *p* = 0.018). On the contrary, the fertility of flies exposed to starting weekly stress was significantly higher on the whole in comparison with the fertility of control flies, despite sharp fertility drops on the exact days of stressing (Figure 1B, stress–F_(1,18)_ = 41.15, *p* ≪ 0.0001).

**Figure 2:**
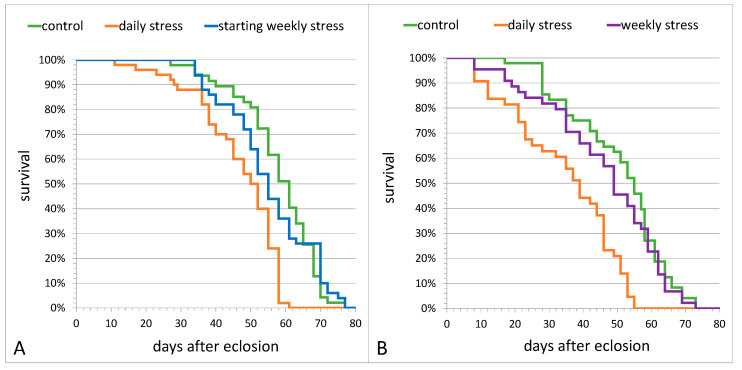
The daily survival rate of *D. melanogaster* Canton S females is affected by mild heat stress (38 °C, 1 h) repeated (**A**) once a day during the overall lifetime (daily stress) or once a week within the first 2 weeks (starting weekly stress); (**B**) once a day or once a week during the overall lifetime (daily stress and weekly stress, respectively). Each group under study includes 50 females.

Next, we tested the effects of short-term heat stress of various frequency on flies’ longevity in comparison with the unstressed control. Control females and females exposed to starting weekly stress had comparable levels of longevity (Figure 2A). In the second experiment (Figure 2B), the longevity of control females and females exposed to weekly stress throughout the whole period of life (until death) was also comparable.

However, the longevity of daily stressed females was significantly lower than the longevity of control females in both experiments (Figure 2A, stress–F_(2,281)_ = 32.90, *p* = 1.5 × 10^−13^; Figure 2B, stress–F_(2,262)_ = 24.91, *p* = 1.3 × 10^−10^; Benjamini–Hochberg post hoc Log-Rank, *p* < 0.0001). The comparison of male longevity in the same groups showed a pattern of stress effect similar to that in females: daily stressed males had lesser lifespans than control flies in both experiments (sex*stress–F_(2,281)_ = 3.04, *p* = 0.050 and sex*stress–F_(2,262)_ = 0.003, *p* = 0.997, correspondingly), while control males had the mean lifespans comparable to that in males exposed to starting weekly stress or weekly stress throughout the whole period of life (Figure S1).

### 3.2. The Effects of Repeated Mild Stress Episodes of Varying Frequency on Appetite, Body Mass, Triglyceride and Carbohydrates Contents

**Figure 3:**
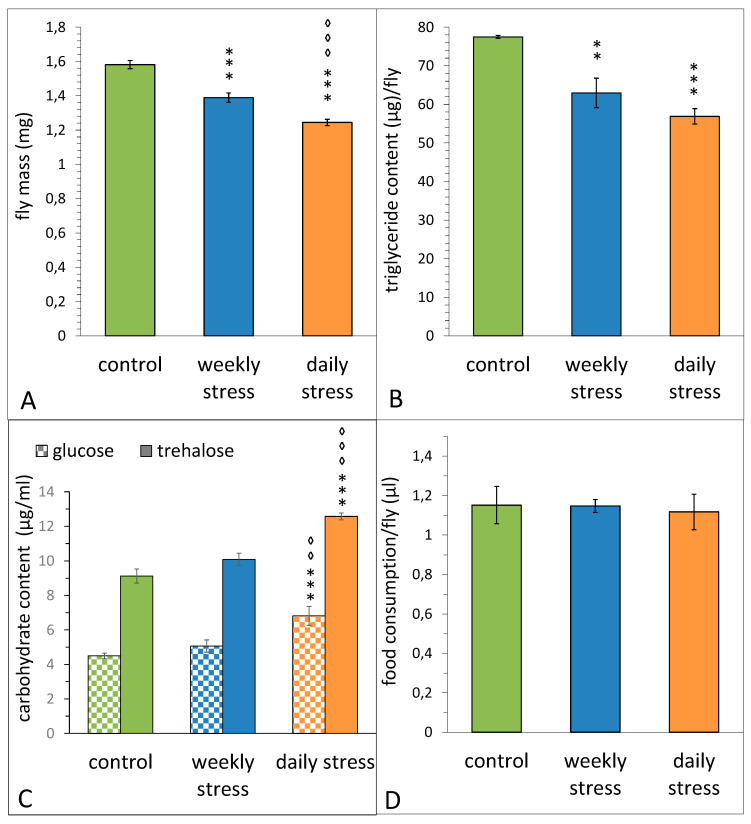
The effect of repeated episodes of mild heat stress (38 °C, 1 h) once a day or once a week within the first 2 weeks on the total mass (**A**), triglyceride content (**B**), carbohydrate content (**C**) and food consumption (**D**) of *D. melanogaster* Canton S females. (**A**) Each value is an average of 9–11 tests. Means ± SEM. (**B**) Each value is an average of 4–6 tests. Means ± SEM. (**C**) Each value is an average of 12 tests. Means ± SEM. (**D**) Each value is an average of 14 tests. Means ± SEM. The asterisk indicates significant differences between stressed and control flies (two asterisks, to *p* < 0.01, three asterisks, to *p* < 0.001, ANOVA test). The diamond indicates significant differences between daily and weekly stressed flies [two diamonds, to *p* < 0.01, three diamonds, to *p* < 0.001, ANOVA test].

Both daily and weekly short-term heat stresses throughout the first two weeks of flies’ lives resulted in a significant weight loss (Figure 3A, STRESS–F_(2,26)_ = 58.53, *p* = 2.4 × 10^−10^). Females exposed to short-term heat stress once a day had a significantly lower body mass compared to females subjected to the same stress exposure once a week.

To estimate body fat composition of the same groups of females, we measured their level of triglycerides. Both daily and weekly stressed females had a significantly lower triglyceride content than control group (Figure 3B, F_(2,26)_ = 18.53, *p* = 0.00022). Taken together, these data suggest that weight difference is connected to body fat percentage of examined flies (Figure 3A,B).

To study the effect of short-term heat stress of varying frequency on carbohydrate metabolism, we measured glucose and trehalose titers in females of the same three groups. Daily stress exposure led to an increase in titers of both carbohydrates, whereas weekly stress did not affect them (Figure 3C, F_(2,33)_ = 21.561, *p* = 0.0000011 for trehalose, F_(2,33)_ = 13.25, *p* = 0.00006 for glucose).

The data of capillary feeding assay (Figure 3D) suggest a redistribution of internal energy resources of the organism under unfavourable conditions, rather than a change in feeding behaviour, as the females of daily and weekly stressed groups do not show any differences in average meal size compared to control females (STRESS F_(2,39)_ = 0.06, *p* = 0.940).

### 3.3. Differentially Expressed Genes (DEGs) Elucidated by RNA-Seq

**Figure 4:**
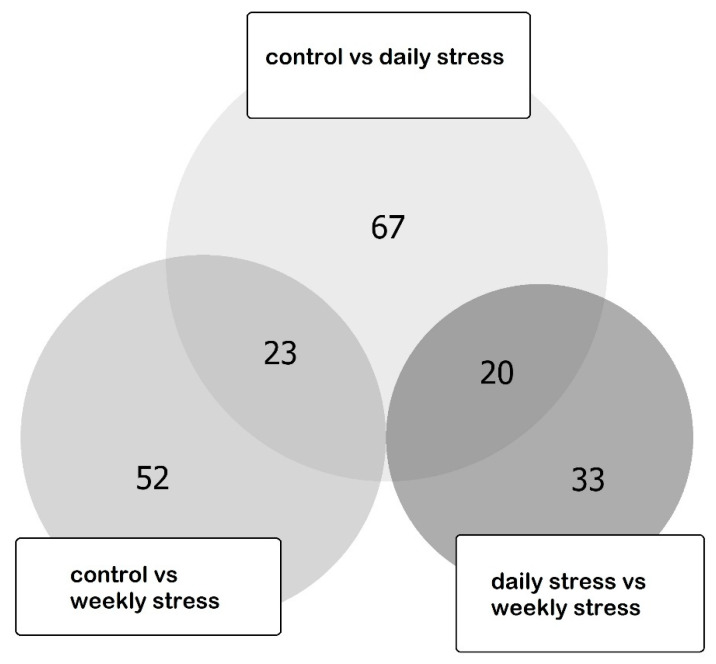
Euler diagram for DEGs in three pairwise comparisons: weekly stressed group vs. control group, daily stressed group vs. control group and daily stressed group vs. weekly stressed group. “Control vs. daily stress” comprises the largest amount of DEGs (67), “control vs. weekly stress” comprises 52 DEGs, and “daily stress vs. weekly stress” comprises 33 DEGs. Overlaps are observed between “control vs. daily stress” and “control vs. weekly stress” (23) and between “control vs. daily stress” and “daily stress vs. weekly stress” (20). No other overlaps are observed.

The pairwise comparison of three groups of species was implemented by Cuffdif utility [38]. We obtained 109 distinct differentially expressed genes, 43 of which were differentially expressed in two pairwise comparisons. Three genes (two long non-coding RNA and one coding) were discarded due to the quite low overall expression rate (total across sample was less than 1 FPKM). The list of differentially expressed genes (DEGs) is presented in Table S1. The DEGs numbers distribution across the comparisons is presented in Figure 4.

### 3.4. DEGs Clustering

**Figure 5:**
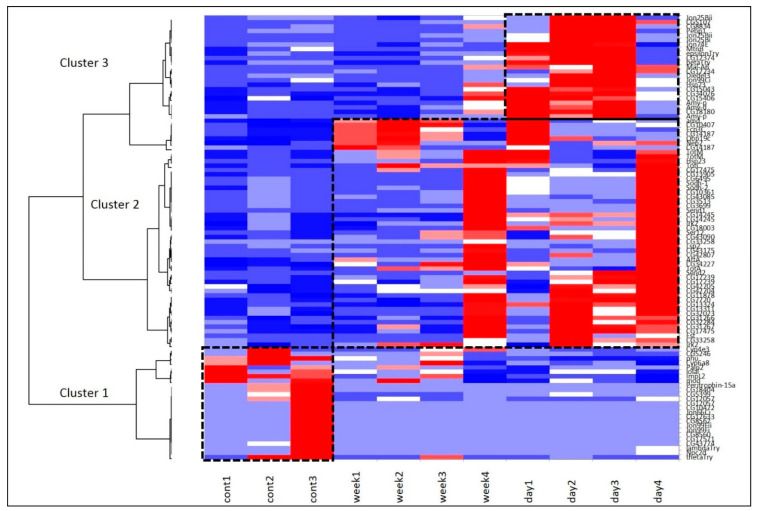
SOM Heatmap based on 109 DEGs underlines three basic clusters. Cluster 1 (bottom left) and cluster 3 (top right) represent the proteolysis gene pathways, and cluster 2 (middle) represents heat shock and *Turandot* gene networks.

We used the same stress modes (stress once a day or once a week) for transcriptome analysis of the fat bodies and adjacent tissues of *D. melanogaster* females. We performed a Self-Organized Map (SOM) procedure resulting in the Heatmap plot presented in Figure 5 and featuring three major gene sets specific for each of the three groups.

To elaborate on the clusters presented in Figure 5, we performed Agglomerative Hierarchical Clustering (AHC; xlstat.com). Three corresponding clusters are presented in Table S2.

### 3.5. DEGs to GO

The next step was to assess specific gene networks by retrieving connected neighbourhoods within each cluster, e.g., to proceed from DEGs clustering to GO annotation. For this purpose, we used the string-db web resource (string-db.org; [39]).

**Figure 6:**
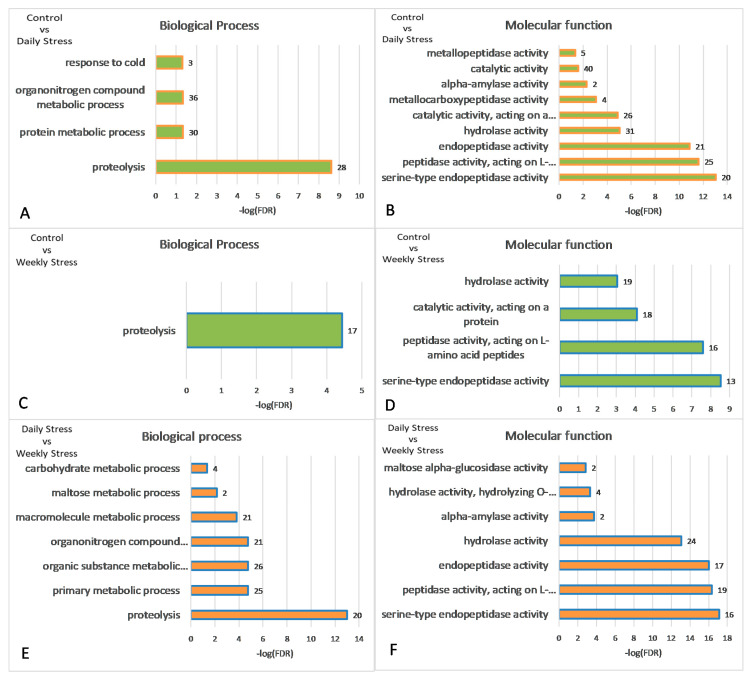
Statistical significance of gene enriched GO categories for pairwise comparison DEGs: (**A**,**B**) control vs. daily stress; (**C**,**D**) control vs. weekly stress; (**E**,**F**) daily vs. weekly stress. Numbers of genes per category are given next to bars. Identifiers of correspondent GO categories can be found in Table S2. −log(FDR = 0.05) = 1.301. All values on the ordinate greater than 1.3 are statistically significant.

**Figure 7:**
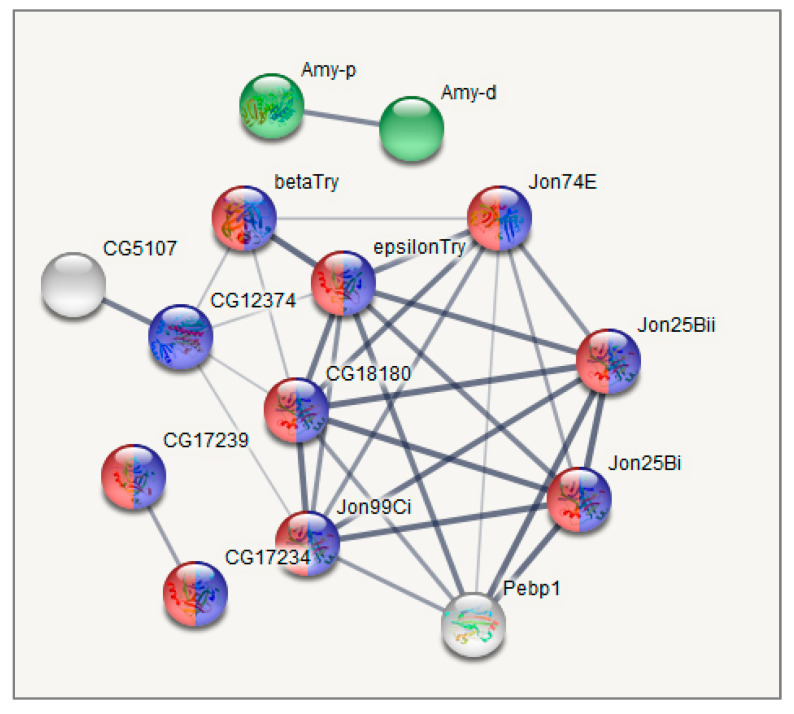
GO enrichment analysis of metabolism related DEGs based on string-db.org database. Cluster 1. Blue: proteolysis (10 genes; enrichment significance over genome average FDR < 10^−5^); Red: serine-type endopeptidase activity (9 Genes; FDR < 9 × 10^−9^); Green: alpha-amylase activity (2 genes FDR < 0.0002). Only connected genes are presented.

**Figure 8:**
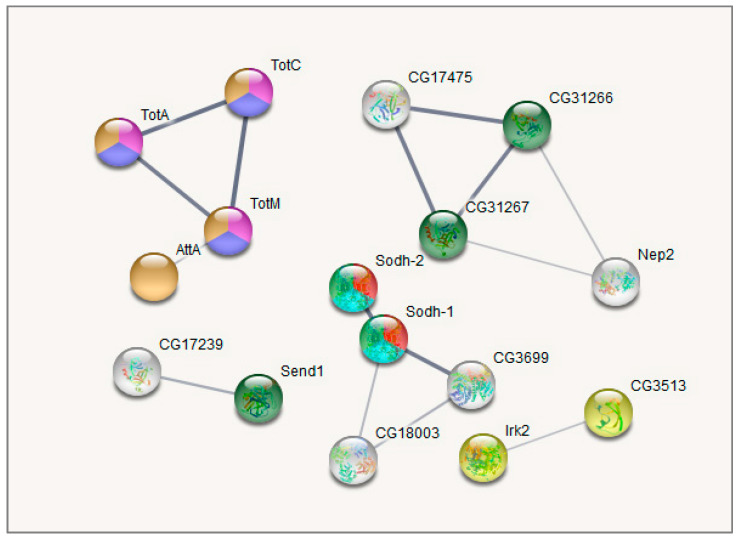
GO enrichment analysis of metabolism-related DEGs along with the correlated heat-stress cluster. Cluster 2. Pink: response to heat (4 Genes; FDR < 0.01; *Hsp23* is not shown); Blue: Stress-inducible humoral factor *Turandot* (3 genes FDR < 0.001); Red: Ketohexokinase, and Glucose dehydrogenase C-terminus (2 Genes; FDR < 0.01); Dark green: including aminoacylase activity, and carboxypeptidase (3 genes FDR < 0.01); Orange: including Innate immunity and *Toll* signalling pathway (4 genes FDR < 0.01); Light green: Alcohol dehydrogenase, zinc-type, conserved site (2 genes FDR < 0.05); Yellow: Fructose and mannose metabolism (2 genes FDR < 0.05); Turquoise: oxidoreductase activity (2 genes FDR < 0.05). Only connected genes are presented.

**Figure 9:**
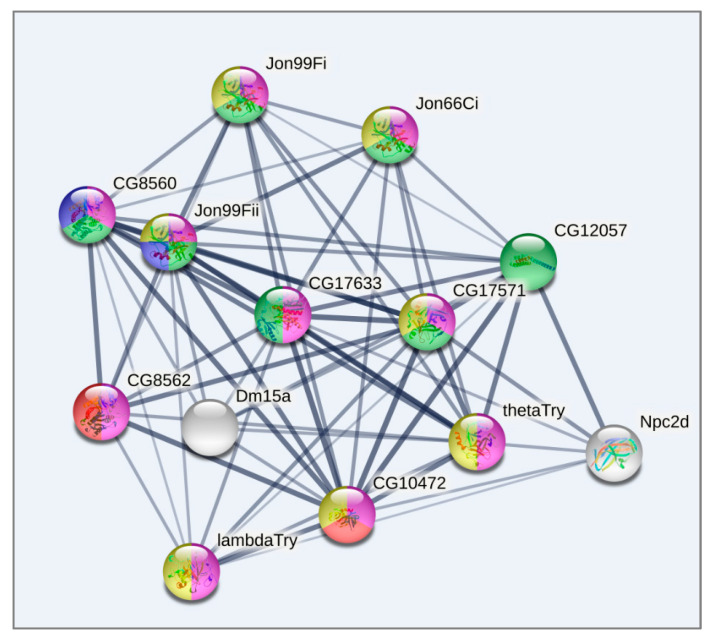
GO enrichment analysis of metabolism related DEGs based on string-db.org database. Cluster 3. Blue: Serine carboxypeptidase, and Trypsin-like peptidase domain (2 genes FDR < 10^−5^); Red: Peptidase S28, and Aminopeptidase N2 (2 Genes; FDR < 10^−5^); Green: Trypsin-like peptidase domain, and carboxypeptidase activity (7 genes FDR < 10^−15^); Yellow: Trypsin-like serine protease (7 genes FDR < 10^−8^); Pink: proteolysis (10 genes FDR < 10^−7^). Only connected genes are presented.

The major GO categories are presented in Figure 6. The full table of enriched GO categories is presented in Table S3. Functional GO enrichment analysis demonstrated significant changes in expression levels of genes involved in metabolic processes following repeated mild heat stress. GO enrichment analysis showed (Table S3, Figure 6, Figure 7, Figure 8 and Figure 9) that GO terms: *alpha-amylase activity* (GO:0004556), *serine-type endopeptidase activity* (GO:0004252), *proteolysis* (GO:0006508), *response to heat* (GO:0009408), *oxidoreductase activity* (GO:0016491), *Stress-inducible humoral factor Turandot* (CL:13062), *Ketohexokinase* and *Glucose dehydrogenase C-terminus* (CL:7048), *including aminoacylase activity* and *Carboxypeptidase* (CL:21109), *including Potassium ion transmembrane transport* and *Aquaporin-like* (CL:15619), *including Innate immunity* and *Toll signalling pathway* (CL:12811), *Serine carboxypeptidase* and *Trypsin-like peptidase domain* (CL:20871), *Trypsin-like peptidase domain* and *carboxypeptidase activity* (CL:20856), *Peptidase S28* and *Aminopeptidase genes* (CL:20920), *Fructose* and *mannose metabolism* (dme00051), *Alcohol dehydrogenase*, *zinc-type*, *conserved site* (IPR002328) and *Trypsin-like serine protease* (SM00020) genes were enriched among 106 differentially expressed genes (DEGs) after multiple test corrections. The stats on GO gene enrichment are presented in Figure 6 and in Table S2.

Next, we proceeded with three clusters of AHC corresponding to three groups to elucidate the major connected neighbourhoods therein.

Cluster 1 (Figure 7), corresponding to the intact metabolome in control group flies, manifests a range of tightly linked proteolysis genes (10 genes FDR < 2.8 × 10^−7^), which mainly consist of genes involved in peptidase activity (9 genes; genes enrichment against whole genome average: FDR < 9.4 × 10^−8^), and two trypsin metabolism genes (*λTrypsin*, *θTrypsin*). The expression of all listed genes is significantly higher in the control group than in the daily and weekly stressed groups (Table S4, Figure 5).

Cluster 2 (Figure 8) contains several groups: genes belonging to (Chymo) trypsin serine peptidase family (7 genes; FDR < 7.4 × 10^−5^) and to response to heat shock (*TotA*, *TotC*, *TotM*, *Hsp23*, *Fst*; FDR < 0.0053) overlapping with three genes from Stress-inducible humoral factor *Turandot* gene network and from *Innate immunity* and *Toll signalling pathway*: *TotM*, *TotC*, *TotA*. The expression of the listed genes is significantly higher in both stress-affected groups than in the control one (Table S4, Figure 5).

Lastly, Cluster 3 (Figure 9), as well as Cluster 1 (Figure 7), contains genes related to proteolysis/digestion (10 genes FDR < 7.4 × 10^−5^), including those reported to be involved in adaptation to chronic nutritional stress ([40]; five genes; FDR < 3.7 × 10^−6^). Notably, their expression level is significantly lower under normal conditions than following daily or weekly stress (Table S4, Figure 5).

Analysing the connected DEGs by means of the string database (string-db.org), we found that the major connected gene network features trypsin domain–containing genes, mostly proteases. The Figure 7, Figure 8 and Figure 9 plots obtained by string-db.org manifest both most significant enrichment and non-random experimentally annotated connections within each cluster. In particular, there are five serine proteases (*αTrypsin*, *βTrypsin*, *εTrypsin*, *λTrypsin* and *θTrypsin*), with a significant shift in expression for the first 3 of them (*αTrypsin*, *βTrypsin* and *εTrypsin*) in daily stressed flies (Table S4, Figure 5), which were featured previously as an adaptation of the nutrition system in the course of chronic stress [40]. These genes also coordinate with α amylases tandem genes network *Amy-P* and *Amy-D* according to clustering.

Another notable gene system that shifted the most in daily interaction is the *Turandot* gene family (shown to be regulated by JAK/STAT signalling pathway, mediating cellular responses to cytokines and growth factors), also featured previously in immune response and stress maintenance [4,41,42] and containing genes *TotM*, *TotA*, *TotC*.

### 3.6. Confirmation of Altered Gene Expression by qRT-PCR

**Figure 10:**
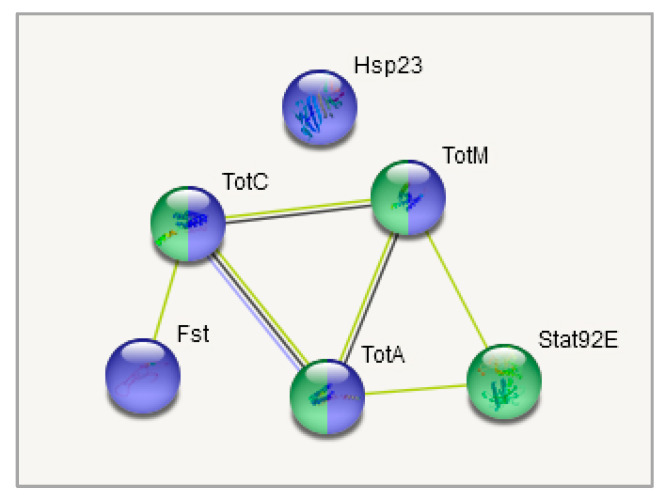
Coexpresion of *Stat92E*, *Fst*, and *TotA*, *TotC*, *TotM* genes relative to samples. Blue: response to temperature stimulus (5 Genes; FDR < 10^−5^); Green: mixed, incl. JAK/STAT pathway, and Stress-inducible humoral factor *Turandot* (4 genes; FDR < 0.001). All of these genes are located in second cluster (Figure 5).

**Figure 11:**
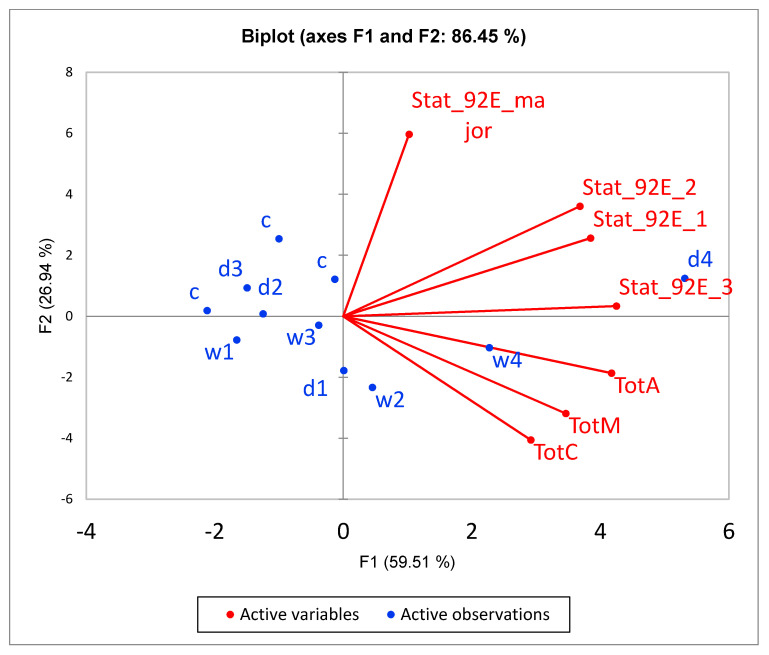
PCA biplot underlines the correlation of *Stat92E* isoforms with *Turandot* family. All *Turandot* genes are significantly correlated within the cluster (*p* < 0.01), and with *Stat_92E_3* isoform (*p* < 0.05).

Four upregulated genes from the DEGs set were chosen for confirmation by qRT-PCR: *TotM*, *TotA*, *TotC* and *Hsp23*. These genes were selected due to connected neighbourhood according to string-db. Enrichment in *Turandot* genes was non-random: both daily- and weekly-stressed groups non-randomly increased their expression compared to the control. Figure 10 demonstrates a coexpresion of *Stat92E*, *Fst*, *Hsp23* and *Turandot* genes. *Stat92E* (*Drosophila* homologue of Signal Transducer and Activator of Transcription protein) gene is known to be involved in generic stress response as a part of the JAK/STAT signalling pathway [43], and *Fst* and *Hsp23* are shown to participate in thermal tolerance [44]. According to Figure 11, the expression of both *Stat92E* and *Turandot* family was burst-like, characterized for stress-response genes. Single representatives from both daily- and weekly-stressed groups significantly increased their expression (Figure 11, Table S5).

**Figure 12:**
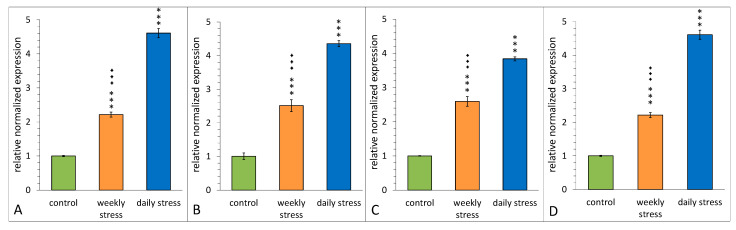
Estimation of relative gene expression, comparing repeatedly stressed flies (weekly stress, daily stress) with flies kept under normal conditions. (**A**)–*TotM*, (**B**)–*TotA*, (**C**)–*TotC*, (**D**)–*Hsp23*. Directionality and relative magnitude of change match microarray data in all cases. We used ANOVA test comparing expression for each gene. Three asterisks–*p* < 0.001 differences from control, three diamonds–*p* < 0.001 differences from daily stress. Error bars show SEM, *n* = 3.

The analysis of RNA extracted from daily stressed, weekly stressed and control flies showed that the qPCR data for all three of them are in agreement with the changes of transcript levels seen in the microarray (Figure 12). The *TotM* expression (Figure 12A) of daily stressed flies was significantly higher than that of control and weekly stressed flies, and the *TotM* expression of weekly stressed flies was significantly higher than that of the control ones (stress–F_(2,6)_ = 290.7, *p* = 1.1 × 10^−6^).

The *TotA* expression (Figure 12B) of daily stressed flies was significantly higher than that of control and weekly stressed flies, and the *TotA* expression of weekly stressed flies was significantly higher than that of the control ones (stress–F_(2,6)_ = 175.7, *p* = 4.8 × 10^−6^).

The *TotC* expression (Figure 12C) of daily stressed flies was significantly higher than that of control and weekly stressed flies, and the *TotC* expression of weekly stressed flies was significantly higher than that of the control ones (stress–F_(2,6)_ = 261.7, *p* = 1.5 × 10^−6^).

The *Hsp23* expression (Figure 12D) of daily stressed flies was significantly higher than that of control and weekly stressed flies, and the *Hsp23* expression of weekly stressed flies was significantly higher than that of the control ones (stress–F_(2,6)_ = 419.9, *p* = 13.6 × 10^−7^).

## 4. Discussion

In this study, we revealed the long-term effects of repeated heat stress on the transcript levels in fly tissues including fat bodies, which are associated with different levels of juvenile hormone metabolism important for reproductive success in female flies. Our findings extended the previous studies on the impact of heat stress on *D. melanogaster* fitness [12,17] and transcriptome [45,46]. In particular, our data complemented the findings of Sørensen et al. [46] concerning stress-induced changes identified in up to 64 h after heat exposure, as well as prolonged stress-induced changes in males [47]. As a result, our data extended the understanding of long-term consequences of cumulative heat stress exposure in insect females.

Herein we found that mild heat stress repeated weekly for two weeks did not affect the longevity, but there was an increase in the total level of fertility, in spite of a sharp fertility drop on the exact days of stressing. Stress once a week throughout the entire period of reproduction did not affect the total level of fertility, although it resulted in a significant fertility decrease on the exact days of stressing. Mild stress repeated daily caused a significant longevity and fertility decrease throughout the entire reproduction period. We believe that this decrease could possibly contribute to adaptation, allowing flies to redirect energy to the fight-or-flight response. This suggestion is supported by the increased resistance of daily stressed flies to acute heat stress [18], as well as by the data on dopamine and energy metabolism ([18]; Figure 3) in these flies. Mild heat stress repeated daily resulted in an increase in activity of dopamine-dependent arylalkylamine N-acetyltransferase and alkaline phosphatase [18], which, together with the decrease in triglyceride levels and the increase in carbohydrate levels in the flies exposed to mild stress once a day (Figure 3B,D), indicates an intensification of dopamine and energy metabolism. Dopamine is known to be involved in the control of many cellular and physiological processes in insects, neuroendocrine stress response and longevity among them [6,48]. This corresponds well with the data obtained on the flies that underwent weekly stress: it does not affect either the dopamine metabolism [18] or the longevity and carbohydrate levels (Figure 2 and Figure 3B). However, such a stressing mode caused a decrease in triglycerides content (Figure 3B). The existence of stress modes that differ in their effect on longevity was previously shown in *Drosophila virilis* [49]. The revealed decrease in triglycerides content following mild heat stress repeated daily or weekly (Figure 3B) also supported the idea of the adaptation process being energy-consuming and corresponds well with the results of Klepsatel et al. [50], who demonstrated a significant effect of heat stress on the fat stores, as well as with our previous data that revealed the decrease in total lipids level in *D. melanogaster* females after acute heat stress [51]. The elevated levels of glucose and trehalose following acute temperature stress (rapid cold hardening) were also shown by Overgaard et al. [52].

To find out which changes in the expression levels of genes were involved in metabolic processes following repeated mild heat stress, we performed whole transcriptome analysis and identified differentially expressed genes in the fat bodies and adjacent tissues of the same groups of *D. melanogaster* females. Here we present novel information regarding upregulated and downregulated genes, pathways and biological processes associated with the adaptation to repeated mild heat stress exposure.

The overall comparison of unstressed control, daily and weekly stressed groups revealed 106 genes to be differentially expressed between control and two stress modes. Most GO terms characteristic for repeated heat stress were related to proteolysis/digestion and response to different types of stress. In general, under repeated mild heat stress exposure, most downregulated genes (Figure 5, cluster 1) are involved in peptidase activity and trypsin metabolism. The DEGs upregulated in daily stressed flies (Figure 5, cluster 3) also belong to the proteolysis gene pathways. To illustrate, cluster 1 contains *θTrypsin* and *λTrypsin* genes, while cluster 3 includes *βTrypsin* and *αTrypsins*. These results suggest changes in the digestion process, as chymotrypsin-like serine proteases are known to be important in protein digestion in insects [53], and do not contradict our data on the decrease in body mass and triglyceride content in stressed flies (Figure 3A,B).

As for the DEGs upregulated in both weekly and daily stressed groups (Figure 5, cluster 2), they mostly represent the heat shock and *Turandot* gene networks.

The absence of heat shock protein (HSP) genes among the differentially expressed genes of both groups under study, which were subjected to repeated stress (with the exception of *Hsp23* in the clusters 2 and 3), is notable. This corresponds to existing data on the development period of heat shock response in *D. melanogaster*: only trace quantities of *Hsp70* were detected after the 24-h recovery period following heat stress [54], and we analysed the flies of experimental groups 24 or 72 h after the last exposure to heat stress. As for *Hsp23*, it is known to be expressed in *D. melanogaster* imago under normal conditions [55]. On the other hand, the *Hsp23* gene expression level was demonstrated to correlate with increased tolerance to different types of stress, including cold, hypoxia, starvation and desiccation [44,56,57]. Here we see that repeated heat stress results in the increase in *Hsp23* expression, which could probably affect the adaptation. The data of Lockwood et al. [58] concerning the ability of *Hsp23* gene overexpression in female ovaries to produce offspring embryos with increased thermal tolerance support this assumption. Moreover, it was shown that muscle-specific overexpression of HSP23 is able to protect muscles from heat stress [59].

Another gene involved in the stress response in *D. melanogaster*, *Frost* (*Fst*), which is induced by cold or heat shock and by desiccation [44,60,61,62,63], was found to be differentially expressed in both daily and weekly stressed groups of flies in our study (Table S2, Figure 5, cluster 2). It is worth noting that *Fst* was shown to be induced only by rather prominent heat stress (38.3 °C for 30 min in [62]) compared to weaker heat exposure, which did not alter *Fst* expression (37 °C for 30 min in [60]; 36 °C for 1 h in [61]). In the present study, we stressed flies at 38 °C for 1 h, which is enough for *Fst* to become involved in the stress response.

Our analysis revealed the increase in mRNA levels following daily and weekly stressing of three genes from *Turandot* family (Table S2, Figure 5 and Figure 7, cluster 2): *Turandot A* (*TotA*), *Turandot C* (*TotC*), *Turandot M* (*TotM*). This family consists of eight genes distributed at three different sites in the *D. melanogaster* genome and could be induced by heat stress and some other adverse impacts [4]. Notably, *TotA* gene also responds to bacterial and fungi infection, cold and oxidative stresses, radiation, wounding and ultraviolet light (UV); *TotC*—to bacterial and fungi infection, cold stress and UV, *TotM*—to bacterial and fungi infection and cold stress [4,43,64,65,66,67]. Conspicuous is the fact that *TotA*, as well as *Fst,* requires rather severe heat stress to induce its transcription and its overexpression has a protective effect on flies against the lethal action of high temperatures [62]. In addition, *TotA*, *TotC* and *TotM* are shown to be regulated by the JAK/STAT (Janus kinase/signal transducer and activator of transcription) pathway in fat body in immune response to septic injury [65,68]. Our data also demonstrate the correlation between *Stat92E_3* isoform and *TotA*, *TotC* and *TotM* expression (Figure 10 and Figure 11). The Jak/STAT pathway in *Drosophila* is important for development and tissue homeostasis; it is shown to be involved in haematopoiesis and cellular proliferation in the gut [68].

Zhong et al. [41] showed that in *D. melanogaster TotM* promotes specific immunity against fungal infection at a cost to flies survival and provides fertility benefits for non-infected females, which corresponds well with our experiments on *Drosophila* fitness under repeated heat stress exposure. The latter upregulates *TotM* expression (Figure 5, cluster 2), and at the same time decreases longevity when applied daily (Figure 2) and increases fertility when applied weekly in the first two weeks (Figure 1). Zhong et al. [41] also suggested that *TotM* could possibly mediate the trade-off between late-age survival and early-age reproduction. The existence of such a trade-off between survival and fitness in *Drosophila* was demonstrated earlier to be the result of multiple cold stress exposure [69].

Notably, our study did not reveal a response to the multiple mild stress of any genes connected with juvenile hormone metabolism, including *Jhe* (*Juvenile hormone esterase*), shown to be expressed in the *Drosophila* fat body [70] and to code the enzyme participating in acute stress response [28]. This fact indicates that the mechanisms involved in the acute stress response differ significantly from those activated by repeated mild stress.

## 5. Conclusions

We found changes in *D. melanogaster* longevity, fertility, metabolism, and the fat body and adjacent tissues transcriptome following two modes of multiple mild heat stress exposure compared to untreated control. We demonstrated that signal pathways related to proteolysis/digestion, as well as the genes of *Turandot* family and two heat stress response genes, *Hsp23* and *Fst*, went through complex regulation in *D. melanogaster* tissues, including fat body under repeated mild heat stress. Moreover, heat stress once a week and once a day resulted in different changes in body composition, fitness and differential gene expression.

## Data Availability

Data have been deposited in the EUROPEAN ENA archive, project PRJEB47414.

## Supplementary Materials

The following are available online at https://www.mdpi.com/article/10.3390/biology10121323/s1, Figure S1: Mean lifespans of *D. melanogaster* Canton S females and males affected by the mild heat stress (38 °C, 1 h). Table S1: Pairwise comparison of q1 (control), q2 (day stress) and q3 (week stress) for significant differential expression (FDR < 0.05) as reported by Cuffdif program, Table S2: Agglomerative hierarchical clustering with consequent merging of sub-clusters to three basic groups depicted in Figure 5, Table S3: GO categories in enrichment of DEGs, Table S4: STAR (Dobin, Gingeras, 2015) summary gene mapping statistics employed on 11 samples, Table S5: Pearson correlation values between 4 *Stat92E* isoforms and *Turandot* family.
