# Supplementary material for: Fitness Analysis and Transcriptome Profiling Following Repeated Mild Heat Stress of Varying Frequency in Drosophila melanogaster Females
Source: Biology (Basel). 2021 Dec 14;10(12):1323. doi: 10.3390/biology10121323 (PMC8698867; doi:10.3390/biology10121323)
Supplement: Supplementary file 1 [file biology-10-01323-s001.zip › Figure S1.pdf]

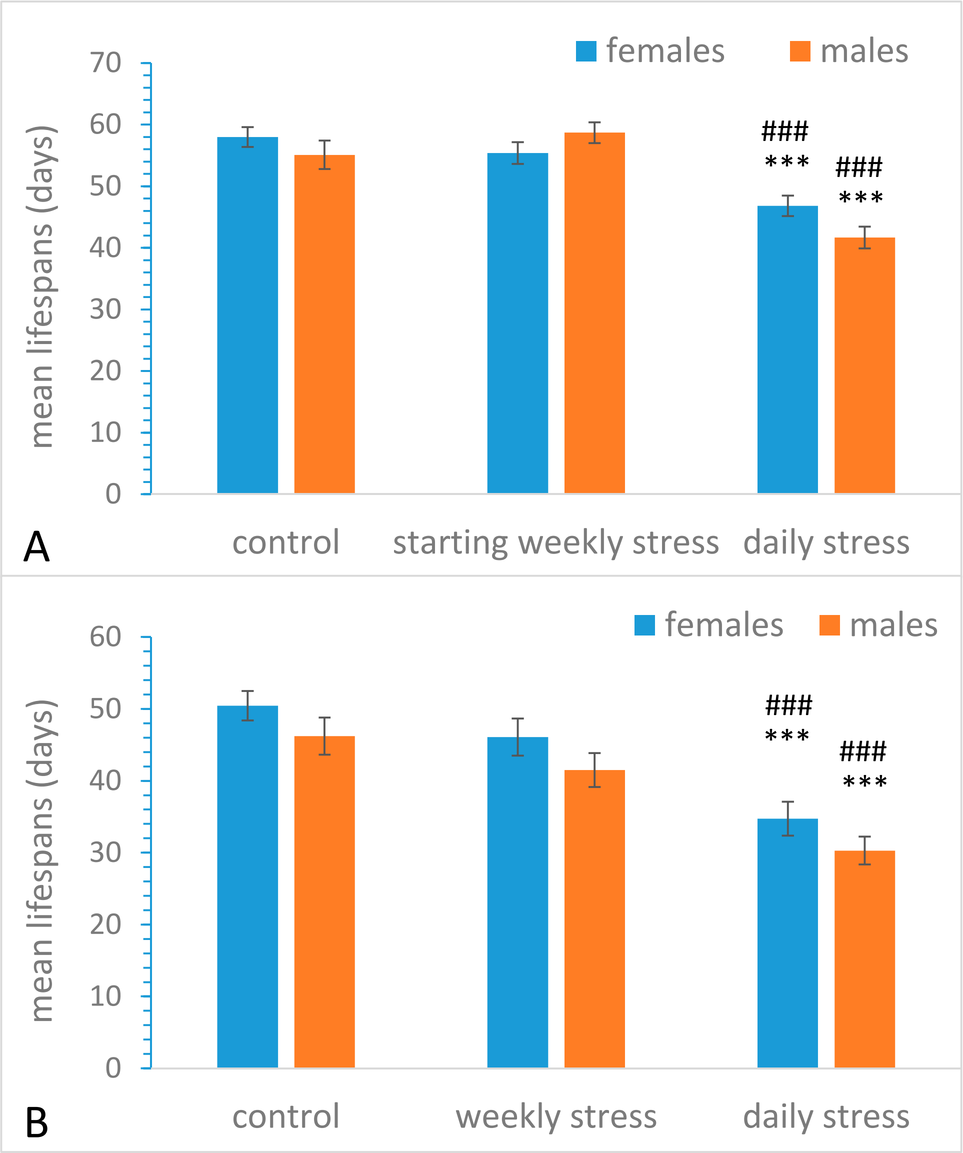

Figure S1. Mean lifespans of *D. melanogaster* Canton S females and males affected by the mild heat stress (38°C, 1 h) repeated (A) once a day during the overall lifetime (daily stress) or once a week within the first 2 weeks (starting weekly stress); (B) once a day or once a week during the overall lifetime (daily stress and weekly stress, respectively). Each group under study includes 50 flies.
