# Supplementary material for: Fitness Analysis and Transcriptome Profiling Following Repeated Mild Heat Stress of Varying Frequency in Drosophila melanogaster Females
Source: Biology (Basel). 2021 Dec 14;10(12):1323. doi: 10.3390/biology10121323 (PMC8698867; doi:10.3390/biology10121323)
Supplement: Supplementary file 1 [file biology-10-01323-s001.zip › Table S2.pdf]

Table S2. Agglomerative hierarchical clustering with consequent merging of sub-clusters to three basic groups depicted in Figure 5. Distinct clusters within one class separated by line.

Control (1)	Day (2)	Week (3)
CG12374	TotM	CG10472
betaTry	Sodh-1	CG12057
Jon25Bi	CG42704	Jon99Fi
CG18180	CG34227	CG18404
Jon25Bii	TotA	Jon99Fii
epsilonTry	CG43090	Peritrophin-15a
Amy-d	AttA	CG8560
Jon99Ci	CG3699	CG17633
Pebp1	CG14245	CG17571
Amy-p	CG42705	Npc2d
MtnB	CG18003	Jon66Ci
CG15406	CG43085	CG43774
Jon74E	Sodh-2	CG5399
CG5107	CG32284	thetaTry
CG34026	CG42807	lambdaTry
Diedel3	CG17239	CG8562
CG15043	CG32023	CG13454
Mal-A6	CG13324	CG5399
CG17234	CG17475	amd
CG8834	CG10361	Obp19c
Irk2	CG43175	CG14187
CG5246	Ser12	Fcp3C
Cyp6a8	CG3513	CG10407
Cyp4e3	CG13905	Nep2
phu	CG6495	Osi2
	Lsp2	
	CG31266	
	CG13311	
	CG7720	
	Send1	
	CG11878	
	Send2	
	CG31267	
	NKCC	
	Hsp23	
	Fst	
	CG33258	
	TotC	
	CG10407	
	ImpL2	
