# Supplementary material for: Fitness Analysis and Transcriptome Profiling Following Repeated Mild Heat Stress of Varying Frequency in Drosophila melanogaster Females
Source: Biology (Basel). 2021 Dec 14;10(12):1323. doi: 10.3390/biology10121323 (PMC8698867; doi:10.3390/biology10121323)
Supplement: Supplementary file 1 [file biology-10-01323-s001.zip › Table S4.pdf]

Table S4. STAR (Dobin, Gingeras, 2015) summary gene mapping statistics employed on 11 samples.

**samples:**	**c1**	**c2**	**c3**	**day1**	**day2**	**day3**	**day4**	**week1**	**week2**	**week3**	**week4**
Number of input reads, mln	38.2	36.2	40.6	34.7	41.5	37.5	40.8	37.5	37.8	32.6	37.5
Average input read length, bp	74	74	73	75	74	75	75	75	74	75	75
**UNIQUE READS:**											
Uniquely mapped reads number, mln	34.9	32.7	36.9	33.0	39.4	35.2	38.7	34.7	35.5	30.7	36.0
Uniquely mapped reads %	91,2	90,2	90,9	95,1	95,0	93,8	94,8	92,5	93,8	94,3	96,1
Average mapped read length, bp	75.3	75.3	75.3	75.3	75.3	75.3	75.3	75.3	75.3	75.3	75.3
Number of splices: Total (thos*)	3894.3	2787.5	3353.0	3774.2	4039.8	3673.1	4799.7	3309.4	3955.4	3182.4	4374.2
Number of splices: Annotated (sjdb**), thos*	3872.4	2766.1	3331.8	3754.0	4018.7	3651.8	4772.9	3289.4	3936.6	3161.9	4352.3
Number of splices: GT/AG , thos*	3862.4	2763.9	3324.4	3747.1	4004.4	3640.7	4764.2	3275.0	3921.7	3155.0	4343.2
Number of splices: GC/AG thos*	31063	22903	27696	26182	34146	31411	34250	33586	32386	26717	30105
Number of splices: AT/AC	839	760	876	944	1211	968	1330	877	1257	675	917

* thos - thousands

** sjdb - splice junctions database is generated from GTF (Dobin A, Gingeras TR. Mapping RNA-seq Reads with STAR. Curr Protoc Bioinformatics. 2015 Sep 3;51:11.14.1-11.14.19. doi: 10.1002/0471250953.bi1114s51. PMID: 26334920; PMCID: PMC4631051.
