# Supplementary material for: Fitness Analysis and Transcriptome Profiling Following Repeated Mild Heat Stress of Varying Frequency in Drosophila melanogaster Females
Source: Biology (Basel). 2021 Dec 14;10(12):1323. doi: 10.3390/biology10121323 (PMC8698867; doi:10.3390/biology10121323)
Supplement: Supplementary file 1 [file biology-10-01323-s001.zip › Table S5.pdf]

Table S5. Pearson correlation values between 4 *Stat92E* isoforms and *Turandot* family.

Variables	Stat_92E_3	Stat_92E_major	Stat_92E_1	Stat_92E_2	TotA	TotC	TotM
Stat_92E_3	**1**	0.251	**0.782**	**0.743**	**0.860**	0.527	**0.672**
Stat_92E_major	0.251	**1**	0.395	**0.615**	0.021	-0.329	-0.212
Stat_92E_1	**0.782**	0.395	**1**	**0.942**	**0.620**	0.282	0.453
Stat_92E_2	**0.743**	**0.615**	**0.942**	**1**	0.566	0.250	0.334
TotA	**0.860**	0.021	**0.620**	0.566	**1**	**0.746**	**0.836**
TotC	0.527	-0.329	0.282	0.250	**0.746**	**1**	**0.639**
TotM	**0.672**	-0.212	0.453	0.334	**0.836**	**0.639**	**1**

Values in bold are different from 0 with a significance level alpha=0.05
